# Multi-omics differences in the bone marrow between essential thrombocythemia and prefibrotic primary myelofibrosis

**DOI:** 10.1007/s10238-024-01350-y

**Published:** 2024-07-08

**Authors:** Anqi Zhang, Ting Sun, Dandan Yu, Rongfeng Fu, Xiaofan Liu, Feng Xue, Wei Liu, Mankai Ju, Xinyue Dai, Huan Dong, Wenjing Gu, Jia Chen, Ying Chi, Huiyuan Li, Wentian Wang, Renchi Yang, Yunfei Chen, Lei Zhang

**Affiliations:** 1 State Key Laboratory of Experimental Hematology, National Clinical Research Center for Blood Diseases, Haihe Laboratory of Cell Ecosystem, Institute of Hematology & Blood Diseases Hospital, Chinese Academy of Medical Sciences & Peking Union Medical College, Tianjin Key Laboratory of Gene Therapy for Blood Diseases, CAMS Key Laboratory of Gene Therapy for Blood Diseases, Tianjin, 300020 China; 2 Tianjin Institutes of Health Science, Tianjin, 301600 China

**Keywords:** Essential thrombocythemia, Prefibrotic primary myelofibrosis, Multi-omics, Proteome, Microbiome, Diagnosis

## Abstract

Essential thrombocythemia (ET) and prefibrotic primary myelofibrosis (pre-PMF) are Philadelphia chromosome-negative myeloproliferative neoplasms. These conditions share overlapping clinical presentations; however, their prognoses differ significantly. Current morphological diagnostic methods lack reliability in subtype differentiation, underlining the need for improved diagnostics. The aim of this study was to investigate the multi-omics alterations in bone marrow biopsies of patients with ET and pre-PMF to improve our understanding of the nuanced diagnostic characteristics of both diseases. We performed proteomic analysis with 4D direct data-independent acquisition and microbiome analysis with 2bRAD-M sequencing technology to identify differential protein and microbe levels between untreated patients with ET and pre-PMF. Laboratory and multi-omics differences were observed between ET and pre-PMF, encompassing diverse pathways, such as lipid metabolism and immune response. The pre-PMF group showed an increased neutrophil-to-lymphocyte ratio and decreased high-density lipoprotein and cholesterol levels. Protein analysis revealed significantly higher CXCR2, CXCR4, and MX1 levels in pre-PMF, while APOC3, APOA4, FABP4, C5, and CFB levels were elevated in ET, with diagnostic accuracy indicated by AUC values ranging from 0.786 to 0.881. Microbiome assessment identified increased levels of Mycobacterium, Xanthobacter, and L1I39 in pre-PMF, whereas Sphingomonas, Brevibacillus, and Pseudomonas_E were significantly decreased, with AUCs for these genera ranging from 0.833 to 0.929. Our study provides preliminary insights into the proteomic and microbiome variations in the bone marrow of patients with ET and pre-PMF, identifying specific proteins and bacterial genera that warrant further investigation as potential diagnostic indicators. These observations contribute to our evolving understanding of the multi-omics variations and possible mechanisms underlying ET and pre-PMF.

**Supplementary Information:**

The online version contains supplementary material available at 10.1007/s10238-024-01350-y.

## Introduction

Essential thrombocythemia (ET) and prefibrotic primary myelofibrosis (pre-PMF) are Philadelphia chromosome-negative (Ph^−^) myeloproliferative neoplasms (MPNs) characterized by clonal myeloid proliferation in bone marrow (BM) [1]. Pre-PMF and ET exhibit similar initial phenotypic features, mainly thrombocytosis [2]. However, these two disease entities have different prognoses [3, 4]. One study indicated that approximately 10% of MPN cases manifest as pre-PMF, which exhibits a lower survival rate than ET [5]. Moreover, two additional studies have shown 15.2% risk of pre-PMF evolving into overt primary myelofibrosis (PMF) and a 4.7% risk of developing into acute myeloid leukemia [6, 7]. The 2016 World Health Organization (WHO) classification also delineated pre-PMF an early indicator preceding significant progression in patients with PMF [1]. Thus, distinguishing pre-PMF from ET is essential owing to their disparate prognoses and therapeutic approaches.

Currently, pre-PMF diagnosis primarily relies on BM histology, complemented by clinical characteristics and driver gene mutations. In the early stages of PMF, the absence of fibrosis, combined with clinical presentations occasionally characterized by isolated thrombocytosis, can misidentify pre-PMF as ET. The precise histological differences between “true” ET and pre-PMF are significant [1]. However, the reproducibility of histological features remains a controversial issue [8]. Overlapping histopathological features and large interobserver variability complicate the distinction of these MPN subtypes [9]. Moreover, some hospitals and institutions lack the specialized personnel and facilities required for making these biopsy diagnoses, necessitating sending biopsies to other institutions. Therefore, it is necessary to identify additional key factors to assist in diagnosing these two disease subtypes.

Several predictive models have been proposed for diagnosing pre-PMF. Carobbio et al. formulated a sequential algorithm incorporating hemoglobin values, leukocyte counts, and serum lactate dehydrogenase (LDH) levels to identify pre-PMF cases that mimic ET [10]. In another study, Schalling et al. [11] proposed a model incorporating splenomegaly alongside laboratory parameters. These studies reflect the unmet clinical need to reliably distinguish between ET and pre-PMF.

Abnormalities in lipid metabolism are common in MPN. A deficiency in serum cholesterol (CHO) is characteristic of MPN [12], with elevated high-density lipoprotein (HDL) levels at diagnosis. However, HDL levels tend to decrease as the disease progresses [13]. Importantly, it is noteworthy that the inflammatory process contributes to symptom onset in MPN and accelerates progression to blast phase MPN or overt MF [14]. A study showed that patients with MPN exhibited higher neutrophil-to-lymphocyte ratio (NLR) compared to the normal population, suggesting a marked inflammatory state [15]. Given the observed abnormalities in lipid metabolism and elevated inflammatory markers in MPN, exploring these clinical indicators may aid in understanding disease mechanisms.

Numerous studies have employed mass spectrometry (MS) proteomics to analyze a range of cancers, including brain [16], gastrointestinal [17], breast [18], lung [19], and liver cancers [20]. These studies demonstrated that proteomic profiles provide supplementary data for patient stratification and facilitate the identification of potential drug targets and disease indicators. Integrating proteomics analysis with other omics datasets could illuminate the molecular mechanisms underlying ET and pre-PMF, facilitating the development of therapeutic agents for these patients.

Traditionally, human blood and bone marrow have been considered sterile environments, prompting microbiome analyses of these tissues only in cases of suspected infections. However, emerging evidence now suggests the presence of a normal blood microbiome in healthy individuals [21]. Meanwhile, growing evidence suggests a connection between human cancers and the microbiome. In hematological malignancies, specific B-cell lymphomas have been linked to the bacterium *Helicobacter pylori* [22]. Additionally, Riquelme et al. emphasized that the microbiome composition of patients with pancreatic adenocarcinoma influences the immune response and disease progression [23]. Recent research has explored the associations between the microbiome and the clinical characteristics of patients with myeloid malignancies, primarily concentrating on the gut microbiome. For instance, intestinal microbiota composition can predict survival outcomes in stem cell transplant recipients [24, 25]. Moreover, one study highlighted a notable variance in fecal microbial community composition between patients with MPN and non-MPN controls, identifying specific species that could potentially contribute to the chronic inflammation of MPN [26]. However, research on the microbiome at the primary tumor site in myeloid malignancies, the bone marrow, remains limited. In addition, the question of whether the bone marrow microbiome could serve as a biomarker for early differential diagnosis between ET and pre-PMF has not been studied.

Therefore, this study aimed to uncover disparities in the proteome and microbiome of BM biopsies from patients diagnosed with pre-PMF and ET. We explored the potential of specific proteins and microbiota to differentiate between ET and pre-PMF, addressing a gap in BM proteomics analysis and microbiome research of these two disease subtypes. Our findings provided preliminary insights into multi-omics variations in the BM of patients with ET and pre-PMF, potentially enhancing our understanding of these conditions.

## Materials and methods

### Patient selection

We included 13 untreated patients diagnosed with ET (*n* = 7, median age: 53 years, m/f = 2/5) or pre-PMF (*n* = 6, median age: 64 years, m/f = 4/2) at the Institute of Hematology and Blood Disease Hospital from October 2016 to February 2023. We obtained formalin-fixed, paraffin-embedded (FFPE) tissue slides of the bone marrow for proteomic and microbiome analysis. The inclusion criteria were: (a) The diagnosis was confirmed based on the 2016 WHO classification [1]. (b) The driver mutation was *JAK2*^*V617F*^. (c) The patients were ≥ 18 years old. (d) The two groups of patients were matched in terms of age, sex, and body mass index (BMI). (e) The patient had not been treated with hydroxyurea, interferons, or other MPN-related cytoreductive drugs. (f) The patient had not used antibiotics, probiotics, prebiotics, or symbiotics in the past three months. (g) The patient did not have autoimmune disease, gastrointestinal disease, any current infection or any other type of cancer except for MPN. Clinical data were retrospectively obtained from the patients’ medical records. Bone marrow biopsies were independently assessed by two experienced hematopathologists who were blinded to the clinical and molecular data of the patients. This study was approved by the Institutional Ethics Committee of the Institute of Hematology and Blood Diseases Hospital and was performed in accordance with the Declaration of Helsinki.

### Proteomic analysis

We utilized the 4D direct DIA method for proteomic analysis. The main steps included sample processing, chromatographic conditions, MS detection, and data analysis. More detailed information can be found in the supplementary material.

### Microbiomics analysis

We used the 2bRAD-M technique for microbiomics analysis. The main steps included DNA extraction, library construction and sequencing, quality control of the sequencing data, identifying species-specific 2bRAD-M markers based on the most comprehensive genome database, and calculation of relative abundance. More detailed information can be found in the supplementary material.

### Functional enrichment analysis

The Gene Ontology (GO) database, established by the Gene Ontology Consortium, enables protein annotation and elucidation of protein functions [27]. The Kyoto Encyclopedia of Genes and Genomes (KEGG) is a comprehensive database regarding biological systems, linking genomic information with functional information in a knowledge base [28]. Here, we used GO and KEGG analyses to further explore differentially expressed proteins (DEPs) functions. PICRUSt2 (Phylogenetic Investigation of Communities by Reconstruction of Unobserved States) is a computational tool designed to forecast functional abundance derived from marker gene sequences [29]. For microbial functional prediction, we used PICRUSt2 (2.3.0b0) software to predict the composition of microbial gene functions based on KEGG Orthology (KO), thereby analyzing functional differences between different groups.

### Protein–protein interaction (PPI) network construction and hub proteins screening

The STRING database (https://string-db.org/) was used to analyze protein interactions [30]. Cytoscape software (version 3.8.2) was utilized to analyze the hub proteins in the PPI network [31]. With the cytoHubba plugin of Cytoscape, the top 10 proteins for each method were determined using four distinct topological analysis techniques (MCC, MNC, Degree, and EPC), and the intersection hub proteins were obtained [32].

### Immune infiltration analysis

CIBERSORT is an analytical instrument capable of estimating the proportions of 22 distinct immune cells [33]. We assessed the infiltration of immune cells in the bone marrow utilizing CIBERSORT.

### Statistical analysis

Statistical evaluations were conducted with R software (Version 4.3.1) [34]. Among the clinical variables, categorical variables were presented as percentages, while continuous variables were denoted as median (interquartile range). Continuous variables were compared between two groups using the Mann–Whitney *U* test.

Alpha diversity (α-diversity) was measured based on species richness and diversity from the rarefied species table. Beta diversity (β-diversity) was determined by calculating the binary Jaccard distance and visualized through principal coordinate analysis (PCoA). Intragroup differences in the α-diversity Chao1 index and PCoA analysis were assessed using the Wilcoxon rank-sum test. Pearson’s correlation coefficient was calculated for quantitative variables with a normal distribution. The correlation heatmap was generated using the ComplexHeatmap package in R software. We employed Circos analysis to delineate the relationship between the samples and species [35]. To identify taxa with differential representation between the two groups, we conducted linear discriminant analysis (LDA) of effect size (LEfSe), setting the threshold for the logarithmic LDA score for discriminative features at greater than 3.0 [36]. To assess the discriminatory potential of notable proteins and microbes, receiver operating characteristic (ROC) curves were plotted, and their AUCs were calculated. A random forest model with cross-validation was also employed to identify significant genera. A two-sided *p* < 0.05 was defined as statistically significant.

## Results

### Differences in clinical and laboratory indicators between ET and pre-PMF

**Fig. 1:**
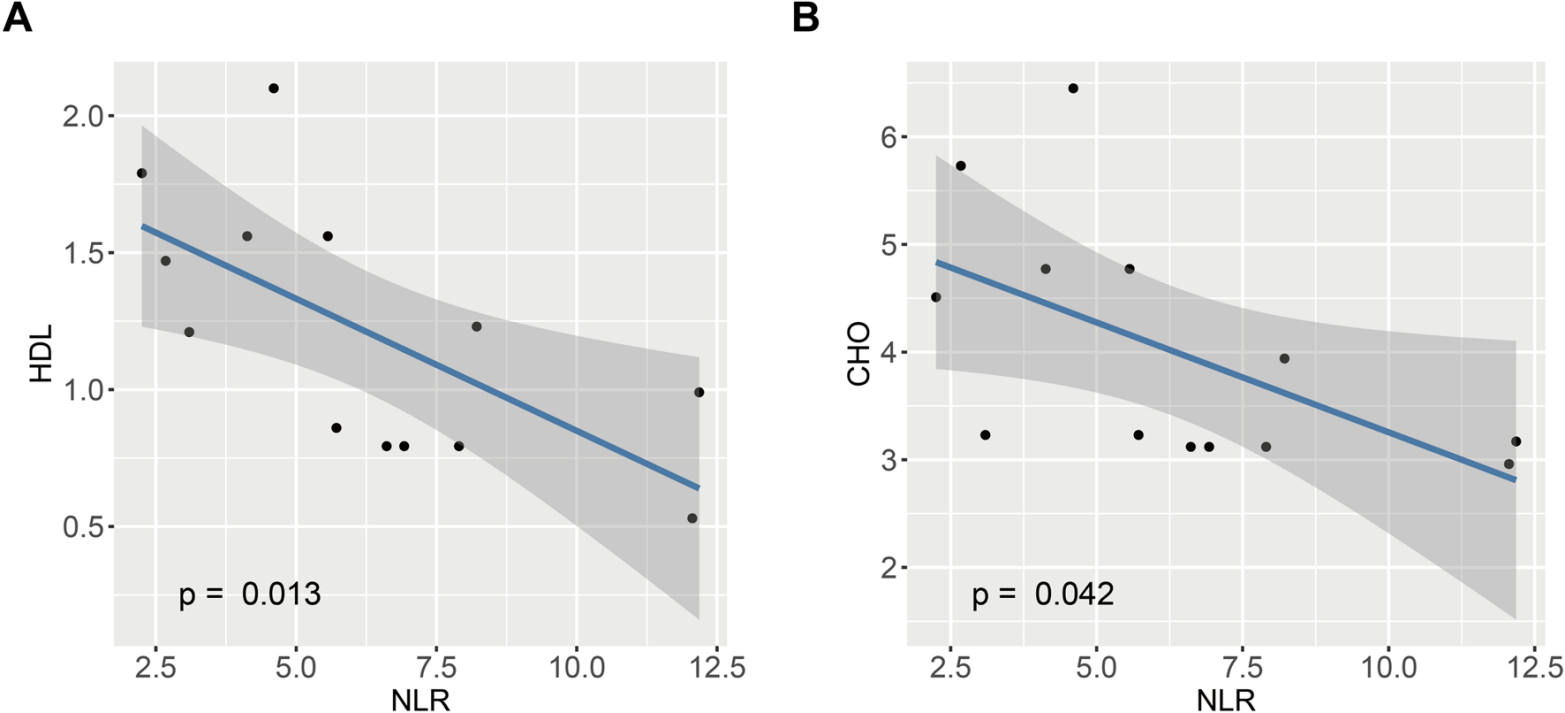
Differences in clinical and laboratory indicators between pre-PMF and ET. **A** Scatter plot of correlation between HDL and NLR. **B** Scatter plot of correlation between CHO and NLR

Table 1 summarizes the clinical and laboratory characteristics of the patients with ET and pre-PMF. The two patient groups included in our analysis were matched for age, sex, and BMI (*p* = 0.100, *p* = 0.286, and *p* = 0.680, respectively). All patients tested positive for *JAK2*^*V617F*^ mutations, with the allele burden significantly higher in patients with pre-PMF than in those with ET (17.9% vs. 55.8%, *p* = 0.001). LDH levels were elevated in the pre-PMF group compared to the ET group (*p* = 0.007). In addition, a statistically significant increase in the NLR, an inflammation-related indicator, was observed in the pre-PMF group compared to the ET group (*p* = 0.015). This finding suggest potential differences in the inflammatory profiles between ET and pre-PMF. HDL levels were significantly lower in the pre-PMF group (*p* < 0.001), while CHO levels were also reduced considerably in pre-PMF patients (*p* = 0.006). These findings indicated a discrepancy in lipid metabolism between ET and pre-PMF. Figure 1A and B shows a significant correlation between NLR and HDL (*p* = 0.013), and a significant correlation between NLR and CHO (*p* = 0.042).

**Table 1:** Clinical, mutation and laboratory characteristics of ET and pre-PMF patients

Characteristic	ET, N = 7	pre-PMF, N = 6	*p* value
Clinical characteristics			
Age, years	53 (33–64)	64 (57–67)	0.100
Female, n (%)	5 (71.0)	2 (33.0)	0.286
BMI	25.7 (21.5–27.8)	23.5 (23.0–24.4)	0.680
*JAK2*^*V617F*^ allele burden	17.9 (15.4–25.4)	55.8 (47.9–59.8)	**0.001**
Laboratory characteristics			
Leukocytes, × 10^9^/L	9.78 (9.21–11.68)	12.25 (11.14–15.12)	0.073
Neutrophils, × 10^9^/L	7.40 (6.55–8.81)	9.91 (9.05–13.13)	0.124
Lymphocytes, × 10^9^/L	2.00 (1.61–2.08)	1.36 (1.25–1.45)	0.078
NLR	4.13 (2.88–5.08)	7.41 (6.69–11.02)	**0.015**
Monocytes, × 10^9^/L	0.32 (0.31–0.42)	0.58 (0.32–0.64)	0.199
Eosinophils, × 10^9^/L	0.14 (0.12–0.25)	0.37 (0.31–0.40)	0.127
Basophils, × 10^9^/L	0.10 (0.08–0.11)	0.08 (0.06–0.19)	0.598
Erythrocytes, × 10^12^/L	4.96 (4.51–5.28)	4.63 (4.27–5.26)	0.869
Hemoglobin, g/L	137 (131–146)	136 (130–137)	0.409
Hematocrit, %	42.3 (41.4–45.0)	41.2(39.6–45.8)	0.661
Platelets, × 10^9^/L	700 (669–869)	956 (554–1073)	0.460
Glucose, mmol/L	4.81 (4.52–5.20)	4.98 (4.91–5.22)	0.636
LDH, U/L	215 (193–236)	327 (285–392)	**0.007**
α-HBDH, U/L	141 (132–149)	213 (175–281)	**0.018**
Triglycerides, mmol/L	0.85 (0.60–1.88)	1.22 (1.21–1.50)	0.662
HDL, mmol/L	1.56 (1.35–1.68)	0.79 (0.79–0.84)	** < 0.001**
LDL, mmol/L	2.55 (2.36–3.61)	1.97 (1.92–2.05)	0.072
CHO, mmol/L	4.77 (4.23, 5.25)	3.12 (3.12, 3.16)	**0.006**
Erythropoietin, mIU/ml	3.15 (2.52–3.94)	1.61 (1.60–2.40)	0.177

Values are presented as Median (interquartile range) or n (%). Bold indicates statistically significant values

*Abbreviations*: ET, essential thrombocythemia; pre-PMF, prefibrotic primary myelofibrosis; MF, myelofibrosis; NLR, neutrophil-to-lymphocyte ratio; LDH, lactate dehydrogenase; α-HBDH, α-hydroxybutyrate dehydrogenase; HDL, high-density lipoprotein cholesterol; LDL, low-density lipoprotein cholesterol; CHO, total cholesterol

### Proteomic analysis of ET and pre-PMF samples

**Fig. 2:**
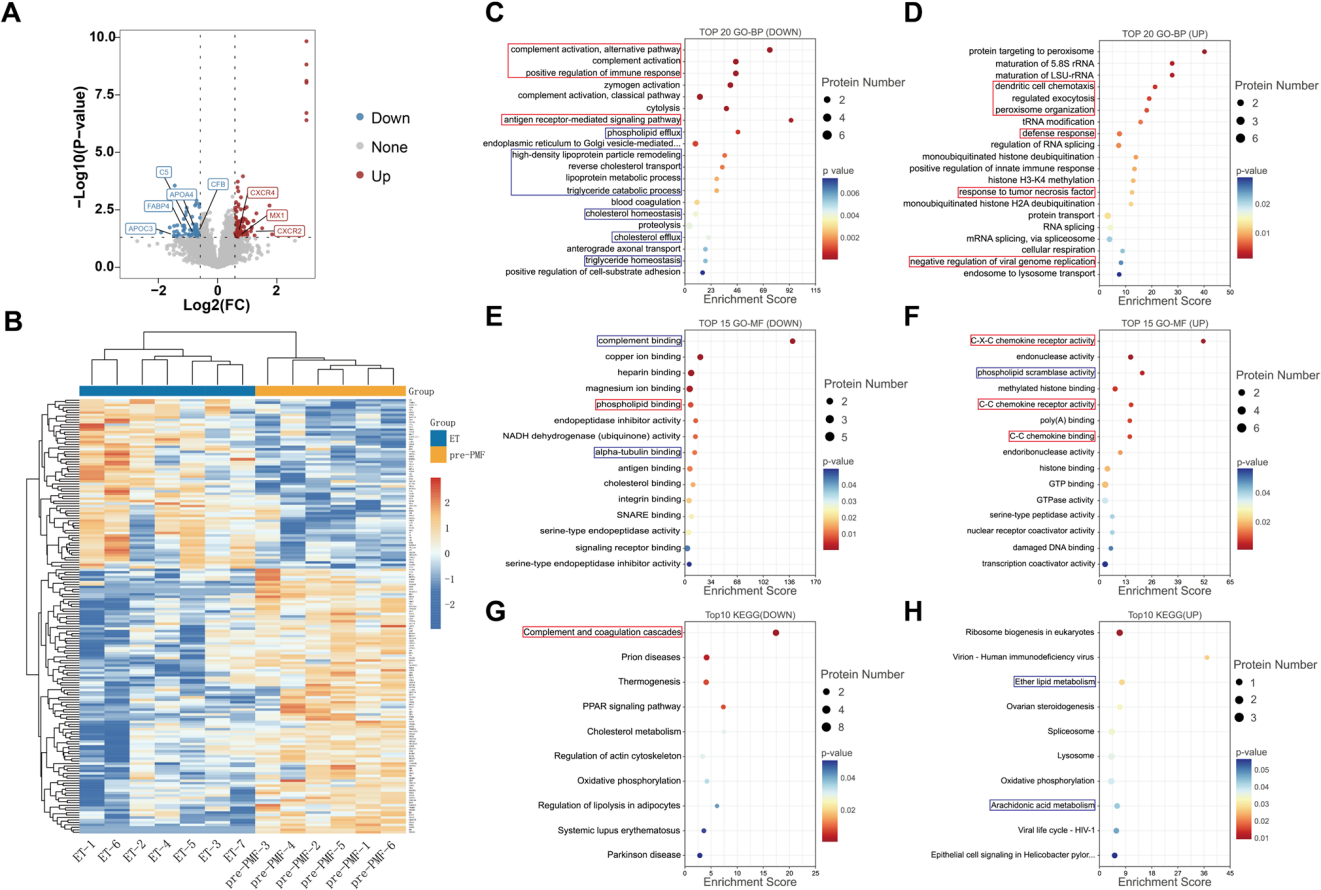
Proteomic analysis of ET and pre-PMF samples. **A** Volcano plots of DEPs with the threshold of |log2(fold change) |≥ 0.585 and *p* value < 0.05. Red points indicate upregulated proteins in pre-PMF, while the blue points denote the downregulated proteins. **B** Heatmap of the 177 DEPs between pre-PMF and ET. Red represents higher expression in pre-PMF, and the blue represents lower expression. Top 20 enriched downregulated (**C**) and upregulated (**D**) GO terms of biological process (BP). Top 15 enriched downregulated (**E**) and upregulated (**F**) GO terms of molecular function (MF). Top 10 enriched downregulated (**G**) and upregulated (**H**) KEGG pathway

**Fig. 3:**
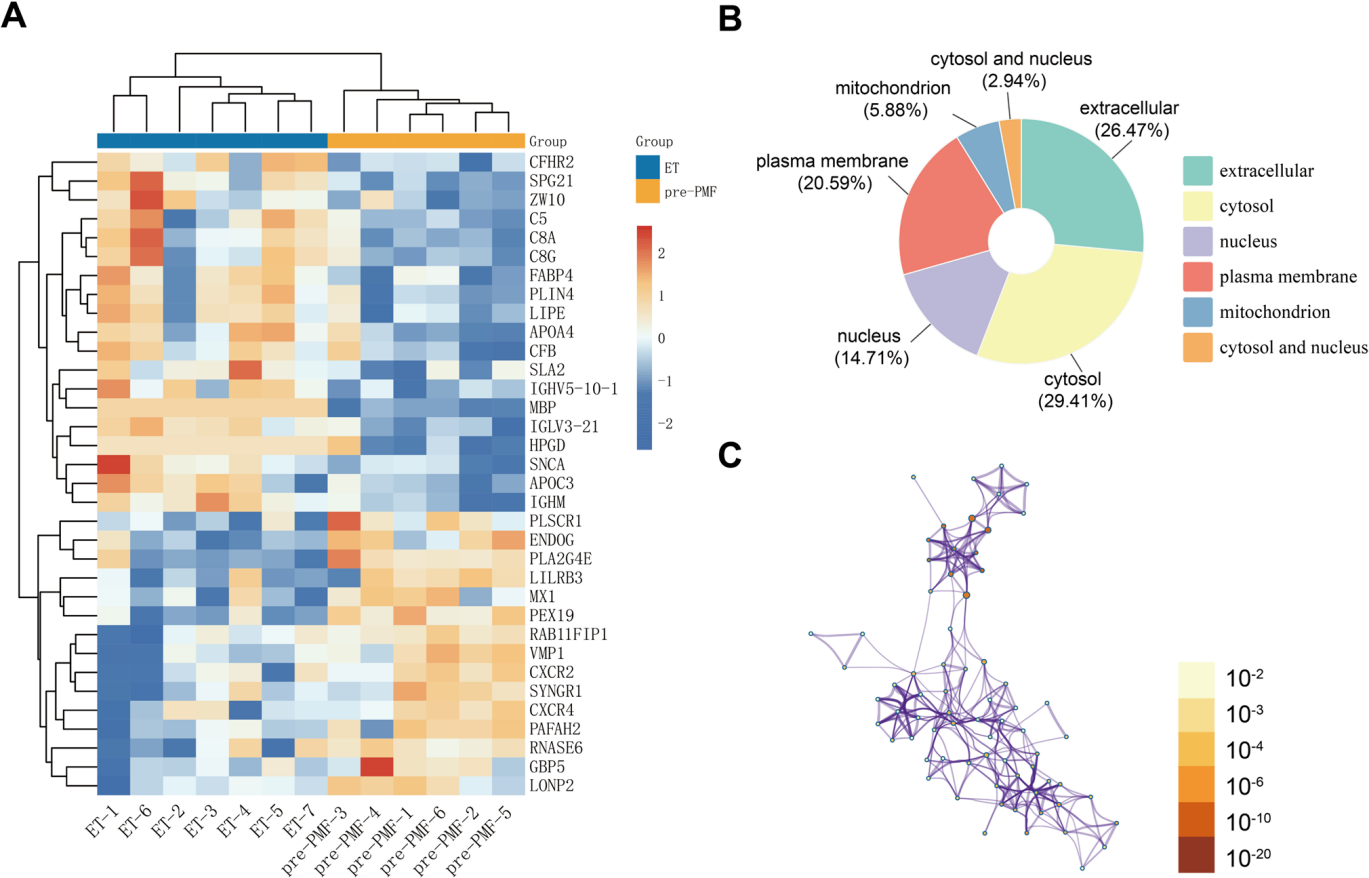
Analysis of 34 DEPs involved in immune response and lipid metabolism. **A** Heatmap of the 34 DEPs involved in immune response and lipid metabolism between pre-PMF and ET. Red represents higher expression in pre-PMF, and the blue represents lower expression. **B** Pie chart of subcellular localization of 34 DEPs. **C** A network of GO-rich terms involved with 34 DEPs colored by *p* value

From the LC–MS/MS analysis of bone marrow paraffin section samples from ET and pre-PMF patients, we discerned a total of 40,786 peptides (Supplementary Fig. S1A) and 5,378 proteins (Supplementary Fig. S1B). DEPs between the two groups were defined based on a fold change (FC) > 1.5 or FC < 0.67, along with *p* < 0.05. Of the 177 DEPs, 102 were upregulated, 69 were downregulated, and six were exclusively detected in pre-PMF (Fig. 2A and B). Subsequently, GO and KEGG pathway enrichment analyses were performed on all DEPs (Fig. 2C–H). The top GO terms (Fig. 2C–F) and KEGG pathways (Fig. 2G–H) were predominantly associated with metabolic processes, inflammatory responses and immune responses. Correspondingly, the enrichment results from the GO and KEGG analyses were consistent with the clinical indicators, wherein the two groups significantly differed in the inflammatory indicator NLR and lipid metabolism-related indicators HDL and cholesterol. Therefore, in our subsequent analysis of the enrichment data, we primarily focused on terms related to inflammation and immunity (highlighted by red boxes) and those related to lipid metabolism (highlighted by blue boxes). We identified 34 DEPs implicated in these processes (Fig. 3A–B). And we displayed the network relationships between the GO terms involved with these 34 DEPs (Fig. 3C). These findings suggest that the changes observed in BM proteins may reflect distinctions in immune response and lipid metabolism between patients with ET and pre-PMF.

### Screening hub proteins through the PPI network

**Fig. 4:**
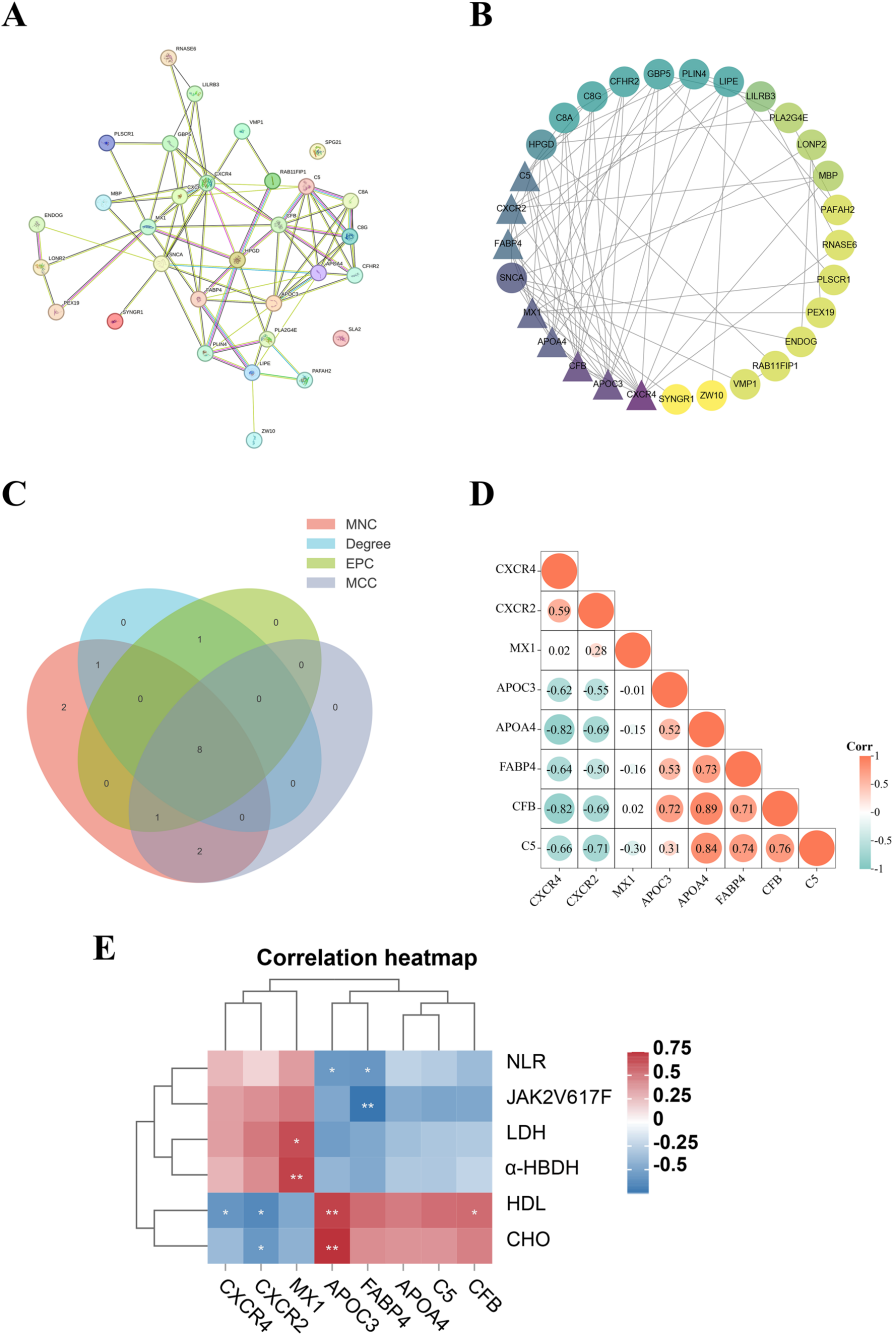
Screening hub proteins through the PPI network. **A** The PPI network of 34 DEPs. **B** Eight statistically significant hub proteins were screened using the Cytoscape software plugin cytoHubba. **C** Intersection of the hub proteins screened using the four algorithms (MNC, Degree, EPC, and MCC). A total of eight hub proteins were obtained. **D** A heatmap of the correlation between multiple proteins and multiple proteins. The horizontal and vertical coordinates represent proteins, and different colors represent correlation coefficients (in the diagram, orange represents positive correlation, green represents negative correlation). **E** Correlation analysis of eight hub proteins and statistically differential laboratory indicators. Red and blue color keys represent positive and negative correlation, respectively. * means correlation 0.01 < *p* < 0.05. ** means correlation 0.001 < *p* < 0.01

The STRING tool was applied to generate a PPI network among the 34 DEPs. It was shown that 31 nodes (proteins) and 77 edges (interactions) were delineated in the constructed PPI network (PPI enrichment *p* < 3.03e−13) (Fig. 4A). Subsequently, utilizing the Cytoscape software, we pinpointed central proteins based on node degrees (Fig. 4B). Eight hub proteins were identified at the intersection of the 34 DEPs, calculated using four cytoHubba algorithms (MCC, MNC, Degree, and EPC), including APOA4, APOC3, FABP4, CFB, C5, CXCR4, CXCR2, and MX1 (Fig. 4C). The correlations between these eight hub proteins are shown in Fig. 4D. Notably, the top two pairs of proteins with intense interactions were lipoprotein APOA4 and the complement-related proteins CFB and C5. Figure 4E shows the correlations between the eight hub proteins and the seven clinically significant indicators. HDL was positively correlated with CFB and APOC3 and negatively correlated with CXCR2 and CXCR4. NLR was negatively correlated with APOC3 and FABP4.

### Association between hub protein expression and immune infiltration

**Fig. 5:**
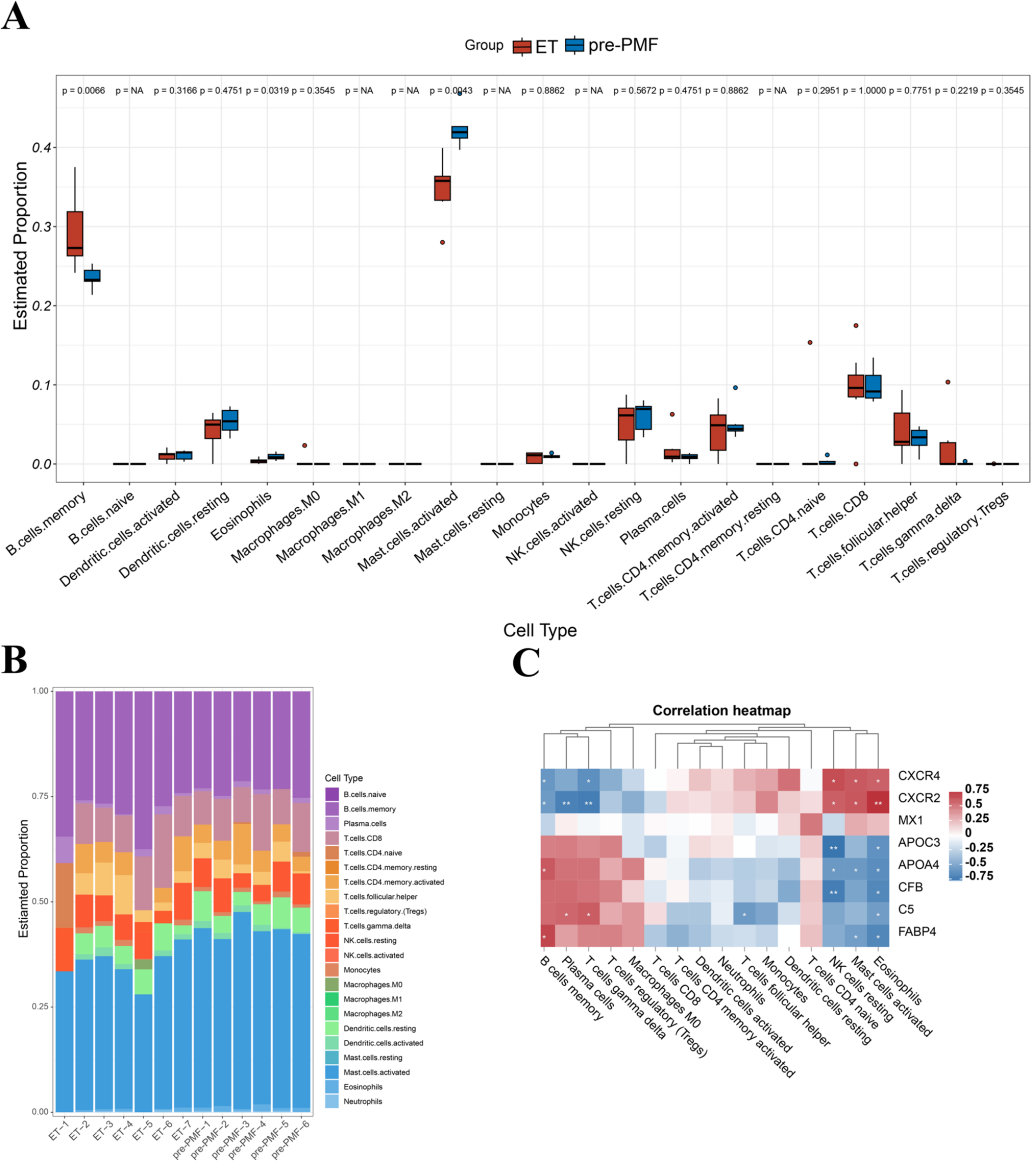
Relationship between hub proteins expression and immune infiltration. **A, B** Differences in immune cell infiltration in pre-PMF and ET samples through CIBERSORT. **C** Correlation analysis of eight hub proteins and immune cells. Red and blue color keys represent positive and negative correlation, respectively. * means correlation 0.01 < *p* < 0.05. ** means correlation 0.001 < *p* < 0.01

The clinical indicators and DEPs analysis indicated a noticeable difference in immune status between ET and pre-PMF bone marrow. To delve deeper, we conducted an immune infiltration analysis using CIBERSORT software (Fig. 5A and B). We found that the innate immune-related cellular components in pre-PMF samples increased compared to ET samples. Specifically, eosinophils and activated mast cells were significantly elevated in pre-PMF samples (*p* = 0.0319, *p* = 0.0043). We extended our analysis to explore the correlation between the eight hub proteins and immune cells (Fig. 5C). Our findings revealed that eosinophils demonstrated a positive correlation with both CXCR4 and CXCR2. At the same time, they exhibited a negative correlation with APOA4, APOC3, CFB, C5 and FABP4. Similarly, activated mast cells showed a positive correlation with CXCR4 and CXCR2 and a negative correlation with APOA4 and FABP4.

### Identification of ET and pre-PMF based on hub proteins

ROC curve analysis was conducted to assess the diagnostic potential of the eight hub proteins in differentiating pre-PMF samples from ET samples. The AUCs for these proteins spanned from 0.786 to 0.881 (Supplementary Fig. S2A–H).

### Variability in microbial diversity and composition between ET and pre-PMF samples

**Fig. 6:**
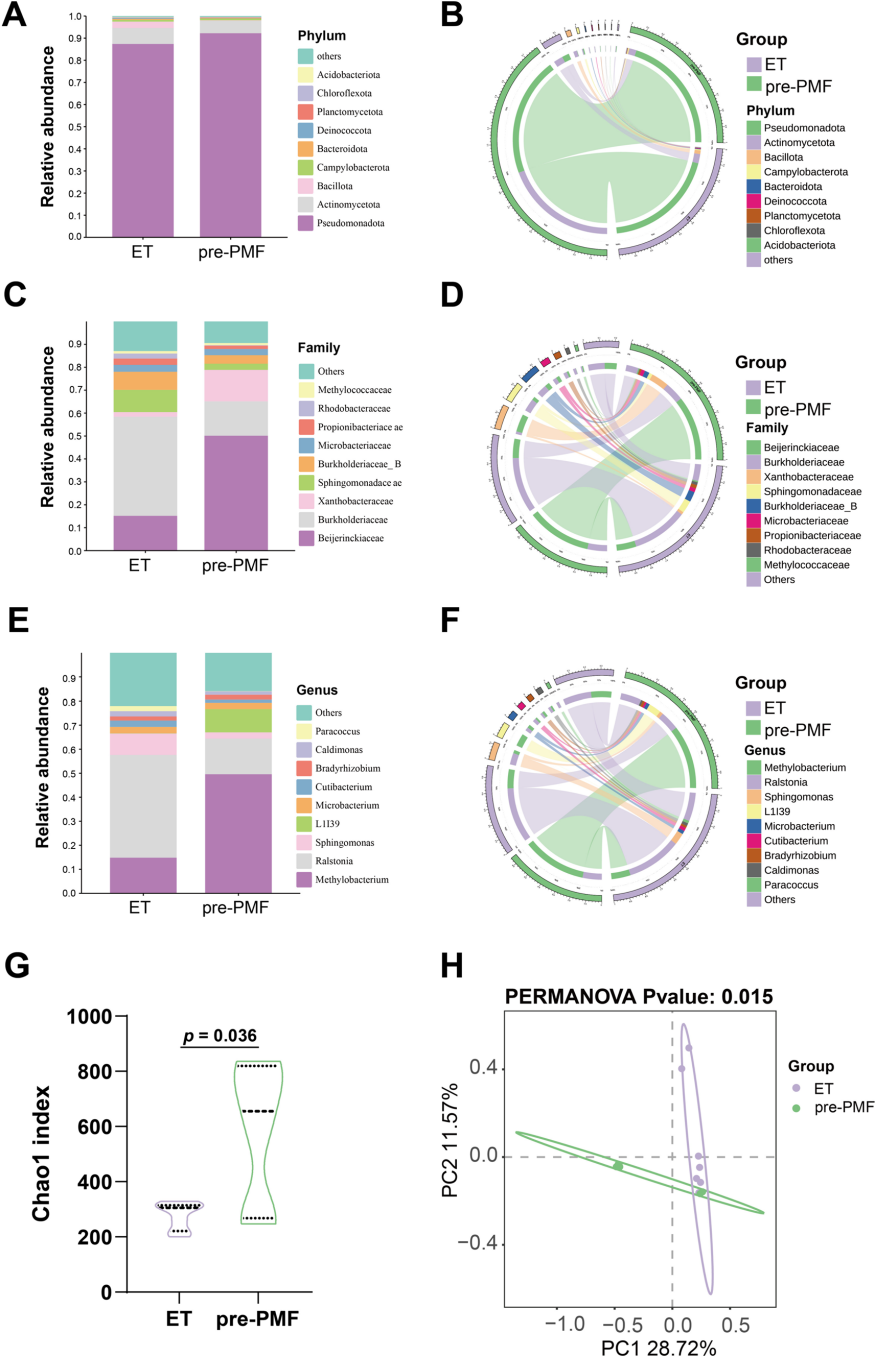
Differences in microbial diversity and composition between pre-PMF and ET samples. The microbial relative abundance of pre-PMF and ET at the phylum (**A, B**), family (**C, D**), and genus (**E, F**) levels. The data are visualized using Circos. **G** Comparison of Chao1 index between pre-PMF and ET. **H** Principal coordinate analysis (PCoA) using binary Jaccard of beta diversity

We further attempted to comprehensively profile the composition of the tissue-resident microbiota by 2bRAD-M. The 2bRAD-M technique possesses the benefits of high sensitivity and high precision. Supplementary Table 1 presents the quality control data acquired during sequencing. From the 13 bone marrow biopsies, we obtained approximately 114 million clean reads, averaging 8.75 million reads per sample. We analyzed the microbiota composition in the ET and pre-PMF samples (Fig. 6A–F). At the phylum level, the top three microbiota of bone marrow were Pseudomonadota, Actinomycetota, and Bacillota. At the family level, the top five microbiota of bone marrow were Beijerinckiaceae, Burkholderiaceae, Xanthobacteraceae, Sphingomonadaceae, and Burkholderiaceae_B. At the genus level, the main microbiota of bone marrow were Methylobacterium, Ralstonia, Sphingomonas, L1I39, Microbacterium, Cutibacterium, Bradyrhizobium, Caldimonas, Paracoccus, and Methylococcus. Notably, the proportions of Ralstonia, Sphingomonas, and Cutibacterium were lower in the pre-PMF than in ET samples (Fig. 6E–F), whereas Methylobacterium and L1I39 were more abundant in the pre-PMF compared to the ET samples (Fig. 6E–F). The Chao1 index in the pre-PMF samples was notably elevated compared to the ET samples (Fig. 6G; *p* = 0.036). PCoA analysis revealed distinct clustering between the pre-PMF and ET tissues (Fig. 6H; *p* = 0.015).

### Significantly different tumor microbiome communities between ET and pre-PMF samples

**Fig. 7:**
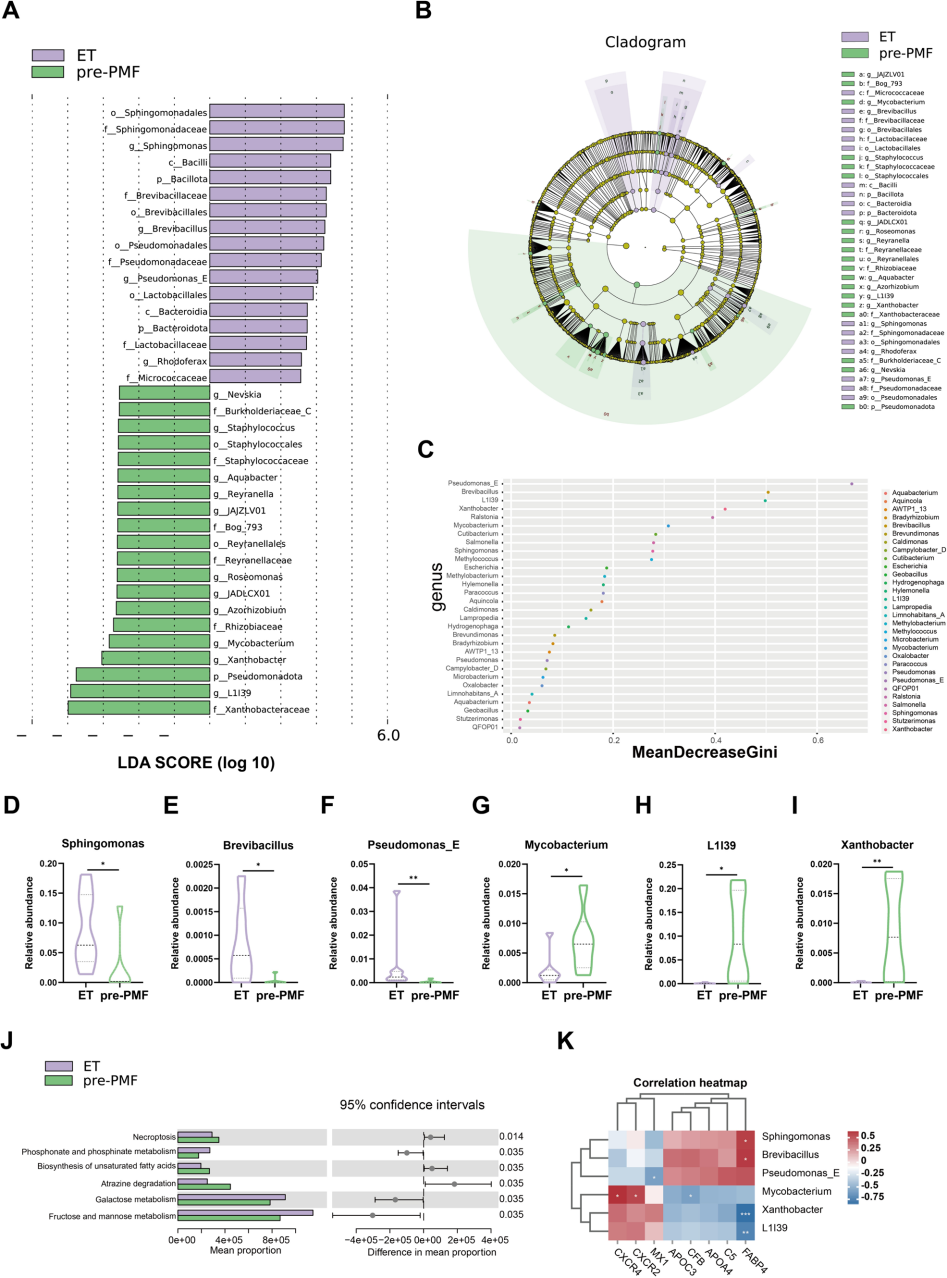
Significantly different tumor microbiome communities between pre-PMF and ET samples. **A** LDA score computed from differentially abundant features between pre-PMF and ET. The criterion for feature selection was log LDA score > 3.0. **B** Taxonomic cladogram from LEfSe, depicting taxonomic associations between microbiome communities from pre-PMF and ET patients. Each node represents a specific taxonomic type. Purple nodes denote taxonomic types more abundant in ET than in pre-PMF, whereas green nodes represent taxonomic types more abundant in pre-PMF. **C** Random forest analysis using the top 30 genus in terms of relative abundance. **D–I** The differential abundance of the six discriminative genera between pre-PMF and ET. **J** The top 6 differential KEGG pathways. **K** Correlation analysis of eight hub proteins and six significantly different genus. Red and blue color keys represent positive and negative correlation, respectively. * means correlation 0.01 < *p* < 0.05. ** means correlation 0.001 < *p* < 0.01. *** means correlation *p* ≤ 0.001

Employing the LEfSe method with a log LDA score threshold of > 3.0, we identified pronounced differences in bacterial community predominance between pre-PMF and ET samples. Specifically, at the genus level, the abundance of Reyranella, Azorhizobium, Mycobacterium, Xanthobacter, and L1I39 was significantly higher in the pre-PMF samples, whereas the abundance of Pseudomonas_E, Brevibacillus, and Sphingomonas was significantly higher in the ET samples (Fig. 7A and B). Additionally, we conducted a random forest analysis using the top 30 genera in terms of relative abundance, obtaining a dot plot of genus importance (Fig. 7C). The random forest analysis confirmed distinct taxa, including Sphingomonas, Brevibacillus, Pseudomonas_E, Mycobacterium, Xanthobacter, and L1I39. Figure 7D–I illustrates the differential abundance of these six distinguishing features at the genus level between the two groups, as assessed by the Wilcoxon test. The abundance levels of these taxa exhibited marked differences between the two groups. We employed KEGG annotation to elucidate the functional alterations within the microbiome of the pre-PMF and ET samples. A total of 7448 KOs were annotated, with 640 showing differential enrichment between patients with pre-PMF and ET. Subsequently, we used PICRUST2 to convert KO abundance to KEGG pathway abundance. Figure 7J shows the top six differential KEGG pathways. We observed an upregulation of the biosynthesis of unsaturated fatty acids pathway in pre-PMF samples (*p* = 0.035). These findings suggest the possibility of an imbalance in lipid metabolism within the BM microbiota. The correlations between the eight hub proteins and six significantly different genera are shown in Fig. 7K. FABP4 exhibited a significant positive correlation with Sphingomonas and Brevibacillus and a significant negative correlation with Xanthobacter and L1I39.

### Identification of ET and pre-PMF based on bone marrow microbiota

ROC curve analysis was performed to assess the diagnostic capability of the six differential taxa to differentiate pre-PMF samples from ET samples. The AUC values for these taxa ranged from 0.833 to 0.929 (Supplementary Fig. S3A–F).

## Discussion

In 2016, the World Health Organization updated the diagnostic criteria for pre-PMF, emphasizing the importance of differential diagnosis between ET and pre-PMF. Furthermore, the distinct prognoses of ET and pre-PMF, along with associated molecular genetic data, bolster the view that they are two separate entities, rather than a continuum of the same disease [1]. Currently, there are methods to separate the two based on clinical and pathological features [37, 38]; however, clinical differential diagnosis can sometimes be challenging, highlighting the need for additional methods to aid in distinguishing between them.

Studies have shown that age [14] and BMI [39] are related to MPN progression. Therefore, we ensured that the two patient groups were matched for age and BMI to minimize potential compounding effects. Furthermore, all included patients harbored the *JAK2*^*V617F*^ driver gene mutation, and none had received MPN-related cytoreductive therapy since the disease onset. Moreover, they had not undergone treatment with antibiotics, probiotics, prebiotics, or symbiotics in the preceding three months, potentially reducing the influence of confounding factors on BM changes. Consistent with a previous study, patients with pre-PMF had a higher *JAK2*^*V617F*^ allele burden than patients with ET [40]. Decreased serum CHO level is a feature of MPN [12]. Research [41] has shown that reduced levels of HDL cholesterol or apolipoprotein A1 correlate with elevated risks of multiple myeloma, MPN, non-Hodgkin lymphoma, and breast, lung, and nervous system cancers. Compared with polycythemia vera or ET, the impact of hypocholesterolemia on the prognosis of MF is more evident [42]. Serum CHO levels have been shown to correlate with the survival rate of PMF [43]. The HDL levels in patients with MPN appear to vary over time; some studies showed that HDL increased at diagnosis [13], and others have found a decrease in disease progression [12, 42]. In our study, we observed significantly lower levels of CHO and HDL in the pre-PMF group compared to the ET group. These findings may have potential implications for prognosis, suggesting further investigation. Our results revealed a significantly higher NLR in patients with pre-PMF than in those with ET. NLR, identified as an inflammatory biomarker in various malignancies [44–46], has been associated with adverse clinical outcomes when elevated. As MPNs are neoplastic disorders characterized by malignant clones triggering inflammatory cytokine release, elevated NLR are commonly observed in patients with MPNs. Some studies [47, 48] have also linked higher NLR values to poorer prognoses within this patient cohort. The elevated NLR observed in patients with pre-PMF in our study suggests a possible rise in systemic inflammatory activity, which may be linked to prognosis. Furthermore, this observation might underscore the potential usefulness of this inflammatory indicator in distinguishing between ET and pre-PMF.

In this study, we analyzed the proteomes of patients with pre-PMF and ET to explore potential differences in protein expression that could aid in distinguishing between the two conditions. We focused on the aforementioned differences in metabolic, inflammatory, and immune clinical indicators in the GO and KEGG top items, highlighting 34 proteins out of 177 that were differentially expressed proteins. We constructed and visualized a PPI network using the STRING database and Cytoscape software. The CytoHubba algorithm was employed to identify the eight hub proteins (APOA4, APOC3, FABP4, CFB, C5, CXCR4, CXCR2 and MX1).

C5, complement component 5, is involved in the complement system. It is cleaved into C5a and C5b. While C5a is vital for chemotaxis, C5b initiates formation of the complement membrane attack complex. CFB, complement factor B, is a component of the alternative complement pathway. Once the alternative pathway is activated, CFB is cleaved by complement factor D, which contributes to the immune response regulation. Activation of the complement system is often observed in autoimmune diseases (e.g., antiphospholipid antibody syndrome [49] and infectious diseases). There is limited research on the complement system in MPN. In our study, the level of complement activation in the pre-PMF group was lower than that in the ET group, possibly because of complement depletion caused by chronic inflammation. One study [50] showed that among patients with myelofibrosis, decreased levels of C3A, C3, and C4 were observed in the most severe cases. Therefore, we hypothesized that a reduction in complement levels could be associated with disease severity and the inflammatory status of the BM. However, further research is needed to validate these hypotheses. APOA4 and APOC3 are apolipoproteins, the primary protein components of lipoproteins. APOA4 is composed of chylomicrons, very low-density lipoproteins (VLDL), and HDL. APOC3 is primarily found in lipoproteins rich in triglycerides, such as chylomicrons, VLDL, and remnant cholesterol. In addition to their crucial role in lipid metabolism, their involvement in tumors has garnered increased attention. Jeong et al. [51] identified reduced APOA4 expression as a marker of cervical squamous cell carcinoma. Research [52] indicated that a reduced concentration of serum APOC3 might assist in the diagnosis of gastric cancer. Our study also observed lower levels of APOA4 and APOC3 in pre-PMF than in ET, implying a potential role for these two lipoproteins in aiding the differentiation between these two disease subtypes. Notably, the top two pairs of proteins exhibiting strong interactions were APOA4 with CFB and APOA4 with C5 (Fig. 4D). CFB demonstrated a significant positive correlation with HDL. Additionally, APOC3 exhibited significant positive correlations with HDL and CHO levels and a significant negative correlation with the NLR (Fig. 4E). These findings suggest a possible connection between lipid metabolism and inflammation. Fatty acid binding protein 4 (FABP4), or adipocyte protein 2, is a carrier protein for fatty acids. It is predominantly expressed in adipocytes and macrophages and is secreted extracellularly. FABP4, when bound to the fatty acid ligand, can interact with unphosphorylated Janus kinase 2 (JAK2), thereby attenuating JAK2 signal transduction [53]. CXCR2, also known as the interleukin eight receptor, beta, is a chemokine receptor. CXCR2 is involved in the migration and activation of neutrophils. It has been reported as an essential component for the recruitment of tumor-associated neutrophils in various cancers [54, 55], playing a notable protumor role. Increased CXCR2 expression is also associated with poor prognosis, as shown by studies on acute myeloid leukemia [56], invasive ductal breast cancer [57], and non-small cell lung cancer [58]. Additionally, Dunbar et al. found that CXCL8/CXCR2 signaling was increased in PMF and that deletion *Cxcr2* in the hMPL^W515L^ model improved blood parameters and decreased fibrosis. This underscores the importance of CXCL8/CXCR2 in fibrosis progression and suggests that inhibiting this pathway could be a promising therapeutic strategy for managing myelofibrosis [59]. In line with prior research, we observed higher CXCR2 expression levels in pre-PMF than in ET, which may suggest a potential association with a more severe prognosis in pre-PMF. CXCR4, or CD184, functions as a chemokine receptor. It plays a role in regulating the migration of various immune cells, such as T cells, B cells, and myeloid cells by interacting with its ligand CXCL12. CXCR4 is commonly found to be highly expressed in hematologic malignancies [60] and many types of solid tumors, including melanoma, and tumors of the lung, breast and ovary [61]. This overexpression is associated with a poorer prognosis [62]. Although Barosi et al. indicated that in patients with PMF, the expression of CXCR4 on CD34 positive blood cells is reduced, with implications for prognosis. This differs from our findings, which showed higher overall CXCR4 protein expression levels in pre-PMF than in ET samples [63]. The discrepancy may stem from differences in the focus of the study, with Barosi et al. concentrating on specific *CD34*-positive blood cells. At the same time, our study encompassed a broader examination of overall protein expression across all cell types in the BM. Additionally, CXCR2 showed a significant negative correlation with the clinical indicators of lipid metabolism, HDL, and CHO levels, while CXCR4 was significantly negatively correlated with HDL. MX1, also known as the interferon-induced GTP-binding protein Mx1, is crucial in inflammation and cancer. Several studies revealed that the level of MX1 protein in the lysates of mononuclear cells from the peripheral blood of patients with systemic lupus erythematosus was markedly elevated compared to the normal controls [64]. Additionally, inhibiting the transcription of *MX1* could alleviate renal fibrosis in lupus nephritis [65]. In cancers such as breast and glioblastoma, MX1 expression was elevated compared to normal tissues [66, 67]. Research on colorectal carcinomas has revealed that the expression level of MX1 in tumors with lymph node metastases (UICC stage III) is higher than that in tumors without lymph node metastases (UICC stage II) [68]. The findings of this study indicated higher MX1 expression levels in pre-PMF than in ET. This suggests a potentially heightened inflammatory state in pre-PMF and raises the possibility of a poorer prognosis in pre-PMF. However, further research is necessary to confirm these associations conclusively.

To further explore the differences in BM immune status between pre-PMF and ET, we utilized CIBERSORT software for the immune infiltration analysis. Our findings showed that pre-PMF has a higher percentage of innate immune cells than ET, with higher proportions of activated mast cells and eosinophils were higher in the pre-PMF group. An increase in the number of eosinophilic granulocytes is occasionally observed in MPNs, and research has also explored their potential protumor effects in other tumors. In preclinical models of oral squamous cell carcinoma, inhibiting eosinophil infiltration led to obstructed carcinoma growth [69]. Similarly, a cervical cancer model indicated that eosinophils activated by tumor thymic stromal lymphopoietin could promote tumor growth [70]. However, the mechanism of eosinophils in MPN requires further investigation. Studies have shown that mast cells play a crucial role in the regulation of inflammatory processes and fibrosis [71]. Activated mast cells produce various substances, including histamines, heparin, tryptases, cytokines, and matrix metalloproteinases, all implicated in fibrogenesis. The proliferation and activation of mast cells are associated with fibrosis in various clinical diseases [72–74]. An increased number of mast cells is observed in MPN samples [75]. Consistently, the increased presence of mast cells in pre-PMF observed in our study may imply a potentially more challenging prognosis for pre-PMF than for ET. Furthermore, we conducted ROC curve analysis for the eight selected hub proteins. Each protein exhibited an AUC value exceeding 0.75, suggesting potential value as biomarkers for differentiating between ET and pre-PMF.

In addition to proteomics, we used 2bRAD-M to characterize BM microbiota in pre-PMF and ET. This method is especially suitable for samples with a low biomass. We found that pre-PMF had higher microbiota diversity than ET. At the family level, the top five microbiota in the bone marrow were Beijerinckiaceae, Burkholderiaceae, and Xanthobacteraceae, whereas the most abundant gut microbiota of MPN [26] encompassed Ruminococcaceae, Lachnospiraceae, and Bacteroidaceae. These results showed that the BM and gut microbiota were different in patients with MPN. Additionally, Andrew Oliver et al. found that the fecal microbial community composition in MPN was related to inflammatory states [26]. This supports the notion that variations in the BM microbiome could potentially serve as markers for differentiating pre-PMF from ET. Using LEfSe and random forest analysis, we identified six genera (Sphingomonas, Brevibacillus, Pseudomonas_E, Mycobacterium, Xanthobacter, and L1I39) that distinguished pre-PMF from ET.

Sphingomonas is a bacterial genus that was subclassified from Pseudomonas approximately 30 years ago. Although it is a Gram-negative bacterium, it contains sphingolipids instead of lipopolysaccharides [76]. It was consistently present in all of our study samples, with abundance notably lower in pre-PMF than ET. In a study on the microbiomics of breast tumors, it was discovered that bacteria belonging to the genera Sphingomonas and the species *Sphingomonas yanoikuyae* were more abundant in healthy breast tissues than in breast tumor tissues [77]. Brevibacillus is a facultative anaerobic, Gram-positive, endospore-forming bacterium that produces a variety of antimicrobial agents including short-sequence microbial peptides as well as glycopeptides, bacilysins, and bacteriocins. Peptides such as Brevilaterin B, extracted from Brevibacillus laterosporus S62–9, are believed to possess anticancer, antibacterial, and antifungal activities [78]. Bogorol is a peptide isolated from *B. laterosporus* JX-5 with potent antibacterial and anticancer activities [79]. According to the Genome Taxonomy Database (GTDB), Pseudomonas_E refers to a specific taxonomic classification within the Pseudomonas genus. The GTDB utilizes genomic data to provide a consistent phylogenetic taxonomy for bacterial and archaeal genomes. Network analysis of 10,000 genomes revealed a phylogenetic tree of Pseudomonas, uncovering at least 14 Pseudomonas groups [80]. Furthermore, Barco et al. used a genomic index (average nucleotide identities and genome alignment fractions) to describe bacterial genera, where Pseudomonas is considered a known polyphyletic genus [81]. Several studies investigating the relationship between *Pseudomonas aeruginosa* and cancer have shown that the blue cupredoxin azurin, secreted by this opportunistic pathogen, enters human cancer cells, leading to apoptosis [82]. Moreover, the presence of detectable *Pseudomonas aeruginosa* and azurin in the tumors of cancer patients has been linked to an increased overall survival rate [83]. Our results indicated that levels of Sphingomonas, Brevibacillus, and Pseudomonas_E were notably lower in pre-PMF than in ET, suggesting a potential contribution to the adverse features in patients with pre-PMF, pending further confirmation. According to the GTDB, both Xanthobacter and L1I39 belong to the bacterial genera of the family Xanthobacteraceae and the order Rhizobiales. Current research on the role of these two bacterial genera in tumors is limited. High-throughput sequencing in microbiome studies has identified a mixed community of Rhizobiales in the lungs and blood of patients with fatal pulmonary illness. Mycobacterium is a genus of over 190 species belonging to the Mycobacteriaceae family. An increasing number of studies have suggested that chronic mycobacterial infections may be associated with an increased cancer risk. This association is primarily attributed to persistent inflammation induced by pathogens. Lung tissues infected with *Mycobacterium tuberculosis* have been shown to undergo multiple cycles of inflammation and tissue repair, creating a favorable environment for tumor formation and increasing the risk of lung cancer [84]. Our study observed an enrichment of the genera Xanthobacter, L1I39, and Mycobacterium in pre-PMF samples. This implies that these bacterial genera are likely associated with pre-PMF pathogenesis, pending further confirmation. Furthermore, we performed ROC curve analysis for the six selected genera, with all AUC values exceeding 0.75. This suggests the potential value of these genera as biomarkers for differentiating between pre-PMF and ET.

The significance of this study lies in its multi-omics analysis of the differences between ET and pre-PMF BM. First, research on the BM proteome and microbiome in these two disease subtypes is rare, and our findings bridge this knowledge gap. Given the growing interest in gut microbiome research, our study underscores the potential importance of investigating the BM microbiome. It provides preliminary insights that could contribute to the broader understanding of the BM microbiome in patients with MPN, suggesting avenues for further research. Second, we utilized the 4D direct DIA and 2bRAD-M techniques for proteomic and microbiomic analyses. Both techniques are particularly suitable for low biomass samples, especially the FFPE of the BM. Finally, our research provided initial insights into variations in the bone marrow proteomics and microbiomes of patients with ET and pre-PMF, pinpointing distinct proteins and bacterial genera that merit additional exploration as potential diagnostic markers.

However, this study has some limitations. First, the sample size is relatively small. While our findings provide promising preliminary insights, they primarily serve as a reference for future research. We hope that subsequent studies can build upon these findings. Second, a prospective study with a more robust methodological design is required to validate and extend our results. Finally, additional comprehensive experiments are necessary to explore the complex interactions between the BM proteome and microbiome, enhancing our understanding of their roles in the pathogenesis and progression of myeloproliferative neoplasms.

## Conclusion

In summary, our study reveals notable disparities in the proteome and microbiome profile of BM between ET and pre-PMF, accompanied by significant alterations in pathways related to lipid metabolism and immune responses. Specifically, proteins CXCR2, CXCR4, and MX1, along with the microbes Mycobacterium, Xanthobacter, and L1I39, were enriched in pre-PMF. In contrast, proteins APOC3, APOA4, FABP4, C5, and CFB, and the microbes Sphingomonas, Brevibacillus, and Pseudomonas_E were decreased in pre-PMF. Our study provides initial insights into bone marrow proteomic and microbiome variations in patients with ET and pre-PMF, identifying specific proteins and bacterial genera that warrant further investigation as potential diagnostic indicators. These findings contribute to our evolving understanding of the multi-omics variations and possible mechanisms underlying ET and pre-PMF.

## Supplementary Information

Below is the link to the electronic supplementary material.

Supplementary file 1 (PDF 763 KB)

## Data Availability

The data that support the findings of this study are available from the corresponding author upon reasonable request.
