# Supplementary material for: Multi-omics differences in the bone marrow between essential thrombocythemia and prefibrotic primary myelofibrosis
Source: Clin Exp Med. 2024 Jul 8;24(1):154. doi: 10.1007/s10238-024-01350-y (PMC11228008; doi:10.1007/s10238-024-01350-y)
Supplement: Supplementary file 1 — (PDF 763 KB) [file 10238_2024_1350_MOESM1_ESM.pdf]

Supplementary Material

Multi-Omics Differences in the Bone Marrow between Essential Thrombocythaemia and Prefibrotic Primary Myelofibrosis

Anqi Zhang, PhD[#1]; Ting Sun, MD[#1]; Dandan Yu, MD[#1]; Rongfeng Fu, MD[1]; Xiaofan Liu, MD[1]; Feng Xue, MD[1]; Wei Liu, MD[1]; Mankai Ju, MD[1]; Xinyue Dai, MD[1]; Huan Dong, MD[1]; Wenjing Gu, MD[1]; Jia Chen, MD[1]; Ying Chi, PhD [1]; Huiyuan Li, PhD [1]; Wentian Wang, PhD [1]; Renchi Yang, MD[1,2]; Yunfei Chen, MD[1*]; Lei Zhang, MD[1,2 *]

[1] State Key Laboratory of Experimental Hematology, National Clinical Research Center for Blood Diseases, Haihe Laboratory of Cell Ecosystem, Institute of Hematology & Blood Diseases Hospital, Chinese Academy of Medical Sciences & Peking Union Medical College, Tianjin Key Laboratory of Gene Therapy for Blood Diseases, CAMS Key Laboratory of Gene Therapy for Blood Diseases, Tianjin 300020, China

[2] Tianjin Institutes of Health Science, Tianjin 301600, China

# Anqi Zhang, Ting Sun and Dandan Yu have contributed equally to this work.

*Corresponding author:

Lei Zhang, State Key Laboratory of Experimental Hematology, National Clinical Research Center for Blood Diseases, Haihe Laboratory of Cell Ecosystem, Institute of Hematology & Blood Diseases Hospital, Chinese Academy of Medical Sciences & Peking Union Medical College, Tianjin Key Laboratory of Gene Therapy for Blood Diseases, CAMS Key Laboratory of Gene Therapy for Blood Diseases, Tianjin 300020, China

Phone: 86(2223)909063

ORCID: 0000-0001-7769-7377

Email: zhanglei1@ihcams.ac.cn

Yunfei Chen, State Key Laboratory of Experimental Hematology, National Clinical

Research Center for Blood Diseases, Haihe Laboratory of Cell Ecosystem, Institute of Hematology & Blood Diseases Hospital, Chinese Academy of Medical Sciences & Peking Union Medical College, Tianjin Key Laboratory of Gene Therapy for Blood Diseases, CAMS Key Laboratory of Gene Therapy for Blood Diseases, Tianjin 300020, China
Email: chenyunfei@ihcams.ac.cn

1. Supplementary Method

1.1 Proteomic analysis

1.1.1 Sample processing

For sample processing, the following steps were undertaken. After deparaffinizing the sample, it was scraped into a centrifuge tube. BT lysis solution, ammonium bicarbonate (ABC) solution, and PMSF were added to cover the sample. Noncontact ultrasonic treatment was conducted at 4°C for 10 min. After ultrasonication, the sample was immediately centrifuged to settle the solution. The samples were heated at 90°C and centrifuged at 800 rpm for 15 minutes, then briefly every 5 minutes to prevent drying. They were cooled quickly to room temperature after heating. Iodoacetamide was added, followed by incubation in the dark at room temperature for 30 minutes. ABC solution was added to achieve a final concentration of BT less than 0.5%. Trypsin and LysC were added for combined enzymatic digestion and oscillated at 37°C and 800 rpm. After digestion, the samples were desalted and subjected to machine testing.

1.1.2 Chromatographic conditions

Mobile phase A: H_2O-FA (99.9%:0.1%, v/v). Mobile phase B: ACN-FA (99.9:0.1, v/v). The sample was separated by being loaded from the autosampler to the analytical column (75 μm×25 cm, 1.6 μm, 120 Å, C18, ionopticks). Gradient elution conditions: 0-45 min, 2%-22% B; 45-50 min, 22%-37% B; 50-55 min, 37%-80% B; 55-60 min, 80% B.

1.1.3 MS detection

For MS detection, the subsequent parameters were employed. The timsTOF Pro2 collected data using the parallel accumulation serial fragmentation (PASEF) DIA mode in the positive ion mode. The capillary voltage was set to 1400 V, the first- and second-stage mass spectrometry scan range being 100-1700 *m*/*z*. The ion mobility window range (1/K0) was 0.7-1.4 Vs/cm^2. The ion accumulation and release time were set to 100 ms, achieving an ion utilization rate close to 100%. A complete PASEF cycle consisted of 10

PASEF secondary stages, with a total cycle time of 1.1 s. In the method settings, a polygonal frame was used to effectively filter out low charge-to-mass ratios and singly charged ions. When selecting the precursor ion, the target intensity of the precursor ion was 20,000, with an intensity threshold of 2,500 set for selection for secondary analysis. In a single 100-ms PASEF, 4 quadrupole isolation windows and 4 tims mobility windows were set, with the quadrupole isolation window being 28 Da. The collision energy varied linearly with ion mobility 1/K0, with fragmentation energies ranging from 20 to 59 eV corresponding to ion mobilities of 1/K0=0.6-1.6Vs/cm^2.

1.1.4 Data analysis

For data analysis, the DIA raw data were processed using Spectronaut Pulsar software, the sequence search file being "UniProt-Homo sapiens-9606-2023.2.1.fasta". Analysis was conducted using the directDIA mode without building a library.

1.2 Microbiomics analysis

1.2.1 DNA extraction

For the extraction of sample DNA, the UPure FFPE Tissue DNA Kit (BioKeystone, Cat. No. M2015-A96) was used to extract genomic DNA from paraffin tissue sections. To assess environmental contamination, five paraffin areas without sample tissue on the edge of the sections were carefully selected using a sterile scalpel, and DNA extracted from them was used for quality control. Agarose gel electrophoresis (1%) was utilized to assess the integrity of the DNA. A Nanodrop 2000 (Thermo) was employed to validate the concentration and purity of the DNA.

1.2.2 Library construction and sequencing

After extracting DNA, we performed library construction and sequencing utilizing the 2bRAD-M technique, closely following Wang et al.'s [1] protocol with slight modifications. We digested DNA, ranging from 1 pg to 200 ng, using 4 U of the BcgI enzyme (NEB) for 3 hours at 37°C. After

digestion, adaptors were attached to the DNA fragments. The ligation process combined 5 µl of the digested DNA with 10 µl of a ligation master mix, comprising 0.2 µM of both adaptors and 800 U of T4 DNA ligase (NEB). The ligation was conducted at 4 ℃ for 12 hours. Subsequently, the ligation products were amplified and analyzed by 8% polyacrylamide gel electrophoresis. Bands of approximately 100bp were isolated from the polyacrylamide gel, and DNA was eluted from the gel over 12 hours at 4°C using nuclease-free water. During PCR, we utilized platform-specific barcode primers to introduce unique barcodes for each sample. Each 20 µl PCR mixture comprised 25 ng of gel-extracted PCR product, 0.2 µM of each primer, 0.3 mM dNTP, 1×Phusion HF buffer, and 0.4 U Phusion high-fidelity DNA polymerase (NEB). PCR products were cleaned with the QIAquick PCR purification kit (Qiagen) and subsequently sequenced on the Illumina Novaseq PE150 platform. The 2bRAD-M procedure was executed at Qingdao OE Biotech Co., Ltd. (Qingdao, China).

1.2.3 Quality control of sequencing data

The raw image data files obtained from high-throughput sequencing were transformed into raw sequencing sequences through base calling analysis. We refer to this as Raw Data. The results were stored in the FASTQ file format, which contained the sequence information of the sequencing reads and their corresponding quality information. Sequencing employed the PE150 strategy. Based on the recognition sites of the experimental enzymes, the reads were scanned and sequences containing the enzyme-cut fragments (called "Enzyme Reads") were extracted. They were then filtered according to the following conditions to generate Clean Reads: (a) Filter out reads where the proportion of unknown bases exceeds 8%. (b) Remove low-quality reads (those where the number of bases with a quality score below 30 exceeds 20% of the total bases in the read) (TableS1).

1.2.4 Identifying species-specific 2bRAD-M markers based on the most

1.2.5 comprehensive genome database

Initially, we acquired 404,199 microbial genomes (encompassing bacteria, fungi, and archaea) from the GTDB and Ensembl databases. Following this, we employed built-in Perl scripts to sample restriction fragments from these microbial genomes using each of the 16 types 2b restriction enzymes. This effort culminated in the creation of a large 2bRAD microbial genome database. 2bRAD tags unique to a species-level taxon, with no overlap with other species, were established as species-specific 2bRAD markers. These markers together constituted the 2bRAD marker database.

1.2.6 Calculation of relative abundance

First, to identify microbial species within each sample, all sequenced 2bRAD tags after quality control were mapped (using a built-in Perl script) against the 2bRAD marker database, which contains all 2bRAD tags theoretically unique to each microbial species in the database. To mitigate the number of false positives in species identification, the G score was calculated for each species detected in a sample. This score represents the harmonic mean between the read coverage of 2bRAD markers belonging to a species and the number of all possible 2bRAD markers of that species. The threshold of the G score for a false positive discovery of microbial species was set to 5 [2].

$$G\ score_{species\ i} = \sqrt{S_i \times t_i}$$

S: the number of reads assigned to all 2bRAD markers belonging to species i within a sample.

t: number of all 2bRAD markers of species i that have been sequenced within a sample.

Subsequently, we computed the mean read coverage for all 2bRAD markers of each species, indicating the number of individuals belonging to a species present in a sample at a given sequencing depth. The relative abundance of a given species was then calculated as the proportion of microbial individuals belonging to a species out of the total number of individuals

from known species that could be detected within a sample.

$$Relative\ abundance_{species\ i} = \frac{S_i/T_i}{\sum_{i=1}^{n} S_i/T_i}$$

S: the number of reads assigned to all 2bRAD markers of species i within a sample.

T: the number of all theoretical 2bRAD markers of species i

2. Supplementary Figure

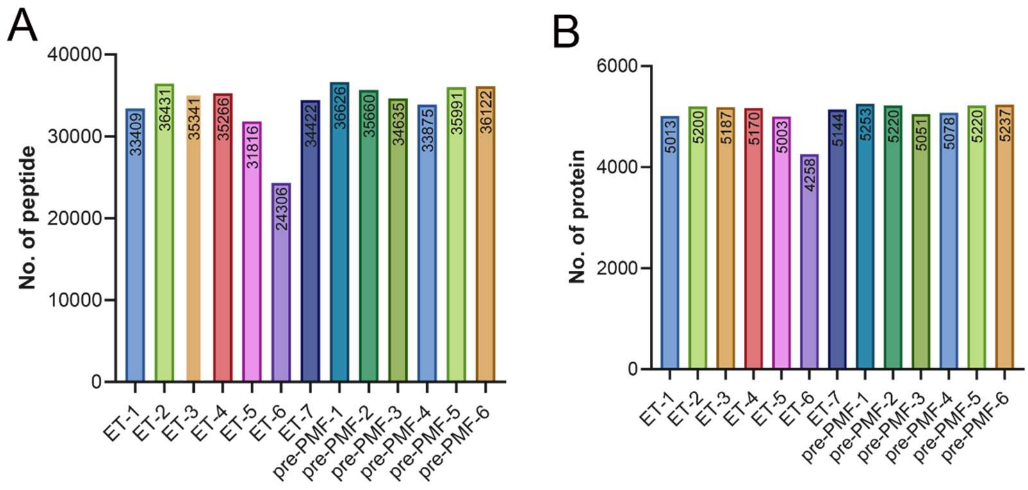

Fig. S1 Quality control. Number of peptides (**A**) and proteins (**B**) detected in every sample

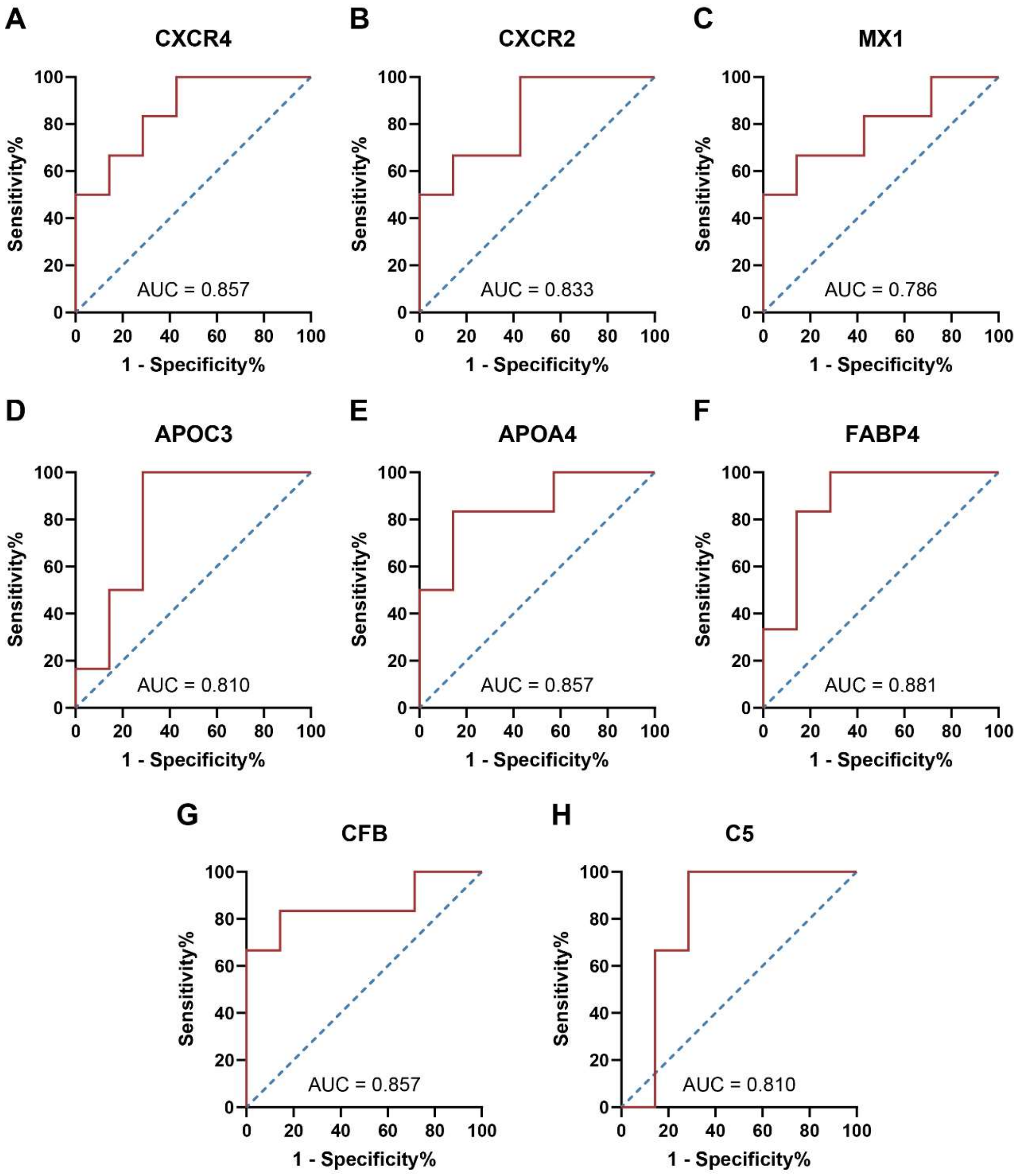

Fig. S2 Identification of ET and pre-PMF based on hub proteins. **A-H** ROC curves for the eight hub proteins.

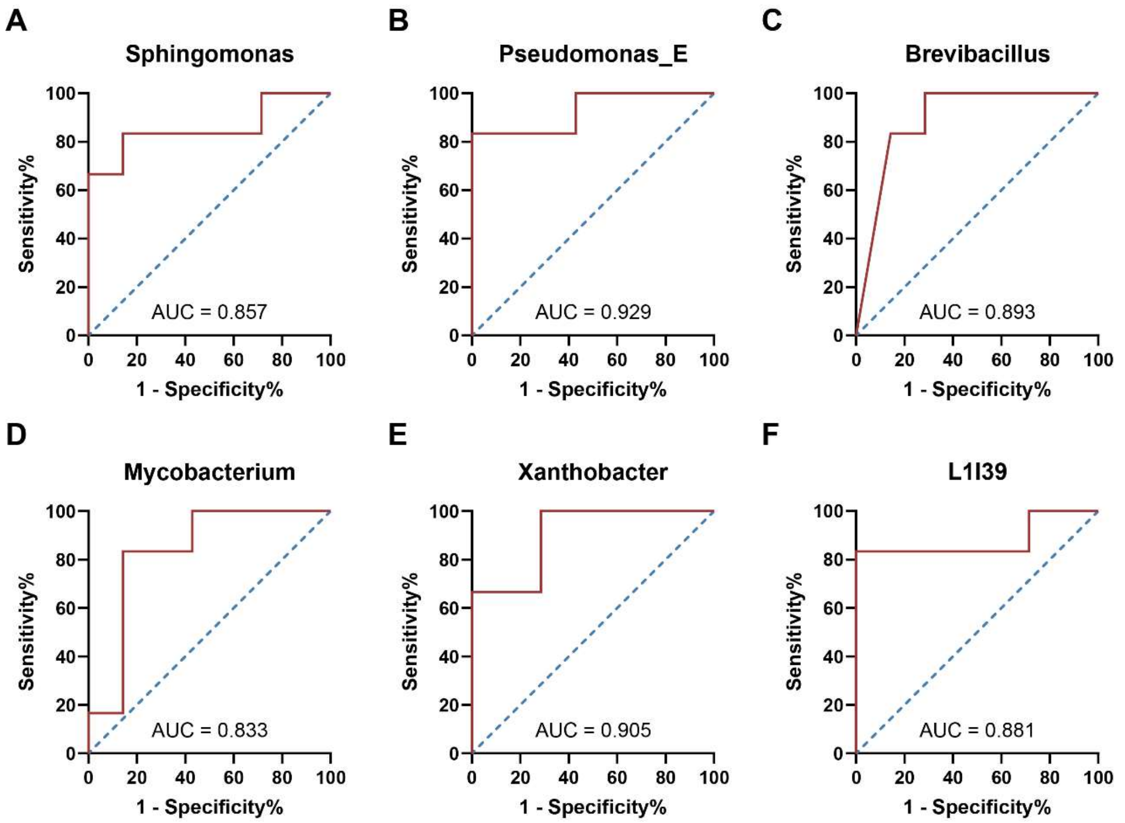

Fig. S3 Identification of ET and pre-PMF based on bone marrow microbiota. **A-F** ROC curves for the six discriminative genera.

3. Supplementary Table

Table S1. Sequencing information summary number of raw reads, enzyme reads, and clean reads and percentage of enzyme reads and clean reads

Sample	Raw reads	Enzyme reads (%)	Clean reads (%)
ET-1	8756073	8243614(94.15)	7844017(89.58)
ET-2	8936461	6933171(77.58)	6667934(74.61)
ET-3	9905698	7906829(79.82)	7443104(75.14)
ET-4	11160372	9551207(85.58)	8989302(80.55)
ET-5	10236006	8625569(84.27)	8101649(79.15)
ET-6	10230238	8605585(84.12)	8097801(79.16)
ET-7	10017640	9049944(90.34)	8555236(85.40)
pre-PMF-1	10898500	10292264(94.44)	9798634(89.91)
pre-PMF-2	11492329	10873744(94.62)	10411996(90.60)
pre-PMF-3	11861654	10863015(91.58)	10450989(88.11)
pre-PMF-4	10767647	10058747(93.42)	9619955(89.34)
pre-PMF-5	9734432	9164179(94.14)	8818041(90.59)
pre-PMF-6	10226445	9433076(92.24)	9016107(88.16)

References

1. Wang S, Meyer E, McKay JK, Matz MV. 2b-RAD: a simple and flexible method for genome-wide genotyping. Nat Methods. 2012;9(8):808-810. https://doi.org/10.1038/nmeth.2023
2. Sun Z, Huang S, Zhu P, et al. Species-resolved sequencing of low-biomass or degraded microbiomes using 2bRAD-M. Genome Biol. 2022;23(1):36. https://doi.org/10.1186/s13059-021-02576-9
